# “Switch-Off-On” Detection of Fe^3+^ and F^−^ Ions Based on Fluorescence Silicon Nanoparticles and Their Application to Food Samples

**DOI:** 10.3390/nano12020213

**Published:** 2022-01-10

**Authors:** Hongli Ye, Lukai Zhao, Xinghui Ren, Youqiong Cai, Hai Chi

**Affiliations:** 1 Laboratory of Aquatic Product Quality, Safety and Processing, East China Sea Fisheries Research Institute, Chinese Academy of Fishery Sciences, Shanghai 200090, China; yehongli12@163.com (H.Y.); caiyouqiong@163.com (Y.C.); 2 Key Laboratory of Control of Safety and Quality for Aquatic Product, Ministry of Agriculture and Rural Affairs, Beijing 100141, China; 3 School of Medical Instrument and Food Engineering, University of Shanghai for Science and Technology, Shanghai 200093, China; 13962892027@163.com; 4 State Key Laboratory of Medicinal Chemical Biology, Tianjin Key Laboratory of Biosensing and Molecular Recognition, Research Center for Analytical Sciences, College of Chemistry, Nankai University, Tianjin 300071, China; 1120200313@mail.nankai.edu.cn

**Keywords:** determination, Fe^3+^ ion, F^−^ ion, fluorescence switch-off-on, silicon nanoparticles, Antarctic krill

## Abstract

An approach to the detection of F^−^ ions in food samples was developed based on a “switch-off-on” fluorescence probe of silicon nanoparticles (SiNPs). The fluorescence of the synthetic SiNPs was gradually quenched in the presence of Fe^3+^ ion and slightly recovered with the addition of F^−^ ion owing to the formation of a stable and colorless ferric fluoride. The fluorescence recovery exhibited a good linear relationship (R^2^ = 0.9992) as the concentration of F^−^ ion increased from 0 to 100 μmol·L^−1^. The detection limit of the established method of F^−^ ion was 0.05 μmol·L^−1^. The recovery experiments confirmed the accuracy and reliability of the proposed method. The ultraviolet–visible spectra, fluorescence decays, and zeta potentials evidenced the fluorescence quenching mechanism involving the electron transfer between the SiNPs and Fe^3+^ ion, while the fluorescence recovery resulted from the formation of ferric fluoride. Finally, SiNPs were successfully applied to detect F^−^ ions in tap water, Antarctic krill, and Antarctic krill powder.

## 1. Introduction

Silicon nanoparticles (SiNPs) have attracted extensive attention in several fields because of their availability in abundance, excellent optical properties, and biocompatibility [1,2,3,4,5,6]. At present, SiNPs feature the metrics of low cost, simple preparation, good water solubility, controllable surface, and high fluorescence quantum yield, which enable them to be environmentally friendly fluorescence probes [7,8,9]. Over the past two decades, tremendous progress has been made in the fluorescence sensor region of SiNPs for intrinsic nontoxicity, which are deemed to be suitable alternatives to traditional II–VI quantum dots [10,11]. Previous works have reported the successful use of SiNPs for the detection of Cu^2+^ [12], Hg^2+^ [13], dopamine [14], heparin [15], 2,4,6-trinitrophenol [16], phosphatase [17], glutathione [18], nitrite [19], chromium [20], cysteine [21], chlorogenic acid [22], and tetracycline [23]. However, there are few studies regarding the use of SiNPs as a probe for the detection of F^−^ ion.

As an essential element, fluorine plays an important role in many aspects of human health, such as dental care and clinical treatment for osteoporosis [24,25]. However, abnormal levels of F^−^ ions can damage aquatic organisms and plants, and lead to several human health issues, including dental health issues, osteoporosis, and acute stomach ulcers [26,27,28]. Therefore, there is a demand for rapid and accurate quantitative methods for the detection of F^−^ ions. Thus far, various determination approaches for F^−^ ions have been developed using the colorimetric method [29,30], ion chromatography [31], gas chromatography [32], and the fluorescence method [33,34]. Among these strategies, the fluorescence method, based on optical probes, has been widely investigated owing to its simplicity, high sensitivity, good selectivity, fast response, and intuitiveness [35,36,37,38,39]. F^−^ ions exhibit the highest charge density, smallest ionic radius, a hard Lewis basic nature, and the most electronegative atom, which enable them to easily combine with metal ions to form the stable compounds [40]. According to this mechanism, many researchers have designed fluorescence sensors to detect the metal ions and F^−^ ions, e.g., Mg [41], Al [42,43,44], Fe-EDTA [45], Ca [46], Hg [47], and Eu [48]. To the best of our knowledge, fluorescence SiNPs have not been used as an environmentally friendly material for the detection of F^−^ ions based on the formation of FeF**^n^**- compounds.

**Scheme 1:**
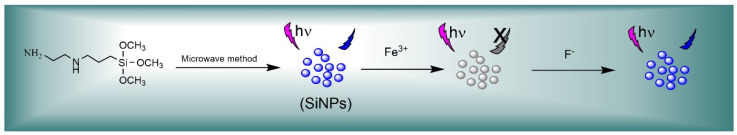
Detection of F^−^ ions based on an off-on fluorescence SiNPs.

Herein, we developed a facile determination approach for F^−^ ions utilizing novel fluorescence SiNPs based on an “off-on” mechanism, as illustrated in Scheme 1. The synthetic SiNPs simultaneously featured bright blue fluorescence with a fluorescence quantum yield of 73.8% and excellent photostability. In a previous study [49], we found that the fluorescence of SiNPs was quenched by Fe^3+^ ion. In this work, we demonstrate that the quenched fluorescence of SiNPs can be restored by the addition of F^−^ ion because of the formation of a stable colorless ferric fluoride. The fluorescence quenching and recovery mechanisms were investigated by using ultraviolet–visible (UV–vis) absorption and fluorescence decay curves. The fluorescence intensity recovery and the F^−^ ion concentrations ranging from 0 to 100 μmol·L^−1^ exhibited a good linear relationship with a correlation coefficient of 0.9992 and a lower limit detection of 0.05 μmol·L^−1^. A recovery assay demonstrated the accuracy and reliability of the developed method for the detection of F^−^ ion in water and food samples.

## 2. Experimental Section

### 2.1. Materials

N-[3-(trimethoxysilyl)propyl]ethylenesiamine (DAMO, ≥99.5%), trisodium citrate dehydrate (SC, ≥99.0%), and glycerol were purchased from J&K Scientific Ltd. (Beijing, China). Trimethylol aminomethane (Tris, ≥99.0%), HCl, NaF, NaCl, NaBr, Na_2_SO_4_, NaNO_3_, NaHCO_3_, Na_3_PO_4_, Na_2_HPO_4_, NaH_2_PO_4_, NaAc, and NaSCN were purchased from Sinopharm Chemical Reagent Co., Ltd. (Shanghai, China). 1,10-Phenanthroline (99%) was provided by Shanghai Aladdin Biochemical Technology Co., Ltd. (Shanghai, China). Fe^3+^ standard solution (1 mg·mL^−1^) and F^−^ standard solution (1 mg mL^−1^) were purchased from the National Non-Ferrous Metal and Electronic Materials Analysis and Testing Center. Ultrapure water was used for all the experiments. All the reagents were used without further purification. The Antarctic krill (*Euphausia superba*) sample was provided by China′s 36th Antarctic Scientific Expedition.

### 2.2. Instruments

The microwave reactor was purchased from Xi’an Yu Hui Instrument Co., Ltd. (Shaanxi, China). A JEM-2100F transmission electron microscope (JEOL, Tokyo, Japan) was used to capture transmission electron microscopy (TEM) images and high-resolution transmission electron microscopy (HRTEM) images at an acceleration voltage of 200 kV. X-ray photoelectron spectroscopy (XPS) was performed using a Thermo Scientific^TM^ K-Alpha^TM+^ (Waltham, MA, USA) spectrometer equipped with a monochromatic Al Kα X-ray source (1486.6 eV) operating at 100 W (Thermo, Waltham, MA, USA). All peaks were calibrated according to the binding energy of the C1s peak (284.8 eV) of adventitious carbon. Inductively coupled plasma emission spectroscopy (ICP-OES) was performed using an iCAP 7000 spectrometer (Thermo, Waltham, MA, USA). It should be noted that the content of SiNPs refers to the Si element not the nanoparticles in this work, which was measured by ICP-OES. The Fourier transform infrared (FTIR) spectrum was recorded using a Nicolet IS5 FTIR absorption spectrophotometer (Thermo, Waltham, MA, USA). A UV-4100 spectrophotometer (Shimadzu, Kyoto, Japan) and an F97Pro fluorescence spectrophotometer (Lingguang, Shanghai, China) were employed to measure the UV–vis absorption spectra and fluorescence spectra, respectively. Fluorescence spectra were measured using an excitation slit of 10 nm, an emission slit of 10 nm, and a gain voltage of 650 V. The relative fluorescence quantum yield (RFQY) was estimated using freshly prepared quinine sulfate in 0.1 mol·L^−1^ H_2_SO_4_ as a contrast. The absolute fluorescence quantum yield (AFQY) was measured using an FS5 fluorescence spectrometer (Edinburgh, UK). The fluorescence decay curves were measured using an FLS-920 fluorescence spectrometer (Edinburgh, UK) equipped with a 405 nm excitation laser.

### 2.3. Preparation of SiNPs

The fluorescence SiNPs were prepared according to a previously reported method [49]. Briefly, 8 mL of glycerol solution was added to a 100 mL flask with argon gas, and 0.3180 g of SC was subsequently added to the Ar-saturated glycerol solution. After the solution was sharply stirred for 20 min, 3 mL of DAMO was added dropwise. The transparent precursor solution was stirred for another 10 min, transferred to an atmospheric microwave reactor, and reacted at 180 °C for 15 min. SiNPs were yielded when the color of the solution was dark brown. Dialysis (molecular weight cut-off: 1000 Da) was performed to remove excess reagents.

### 2.4. Fluorescence Assay with F^−^ Ions

50 μL of 44.08 mg·L^−1^ SiNPs solution, 45 μL of 10 mmol·L^−1^ Fe^3+^ standard solutions, and a series of different volumes of F^−^ standard solutions were added to the centrifugal tubes. Tris-HCl (pH 7.2) reagent was used to fix the final volume to 1.5 mL. The ultimate concentrations of SiNPs and Fe^3+^ ions were 1.5 and 300 μmol·L^−1^, respectively. After mixing for 1 min and standing for another 10 min, the fluorescence emission spectra of the solutions were measured at an excitation wavelength of 370 nm with a fluorescent cuvette. Three separate parallel replicates were used for each experiment.

### 2.5. Anion Selectivity Assays

Stock anion solutions of F^−^, Cl^−^, Br^−^, SO_4_^2−^, NO_3_^−^, HCO_3_^−^, PO_4_^3−^, HPO_4_^2−^, H_2_PO_4_^−^, Ac^−^ and SCN^-^ were prepared by dissolving their sodium compounds in deionized water. A series of 50 μL of 44.08 mg·L^−1^ SiNPs solution, 45 μL of 10 mmol·L^−1^ Fe^3+^ standard solution, and the aforementioned stock anion solutions were added to the centrifugal tubes. Tris-HCl (pH 7.2) reagent was used to fix the final volume to 1.5 mL. The ultimate concentrations of SiNPs, Fe^3+^, and anions were 1.5 mg·L^−1^, 300 μmol·L^−1^, and 1 mmol·L^−1^, respectively. After stirring for 1 min and standing for another 10 min, the mixed solutions were subjected to fluorescence measurements at an excitation wavelength of 370 nm. Three separate parallel replicates were used for each experiment.

### 2.6. Detection of F^−^ Ions in Food Samples

1.00 ± 0.02 g of the homogenized samples (i.e., Antarctic krill and Antarctic krill powder) was accurately weighed in the centrifugal tubes. A certain volume of H_2_O (20 mL for the Antarctic krill and 60 mL for the Antarctic krill powder) was added to the samples. After stirring for 1 min, the reactions were subjected to ultrasonic extraction for 30 min and centrifuged for 10 min at a speed of 10,000 rpm. After filtration through a 0.22 μm microporous membrane, the solution was diluted 15-fold for further measurements.

## 3. Results and Discussion

### 3.1. Structures of SiNPs

**Figure 1:**
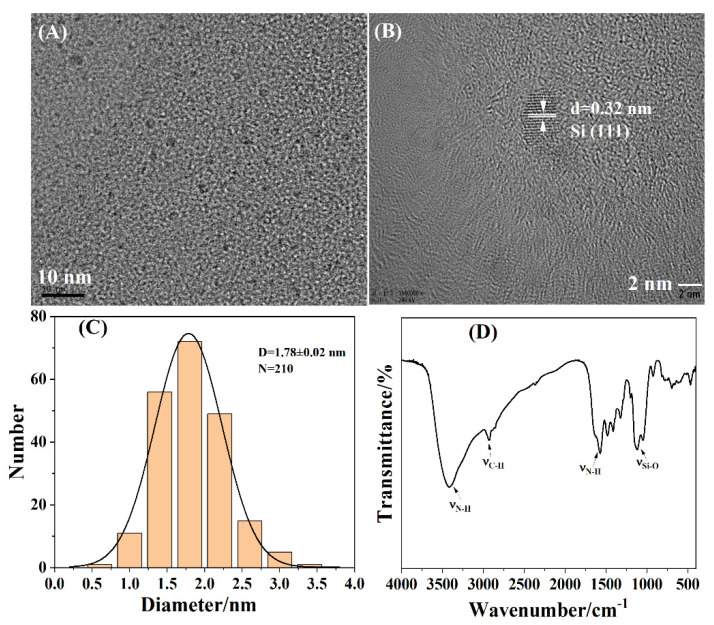
Structural characterizations of the as-prepared SiNPs: (**A**) TEM images; (**B**) HRTEM images; (**C**) size distribution histogram; (**D**) FTIR spectrum.

Figure 1 displays the structures of the as-prepared SiNPs. As observed in Figure 1A, the SiNPs appeared as monodispersed, spherical particles. The HRTEM image (Figure 1B) revealed that the lattice distance of the as-prepared SiNPs was 0.32 nm, which was attributed to the Si (111) crystal plane. The size distribution histogram in Figure 1C shows that the average size of SiNPs was 1.78 ± 0.02 nm (N = 210). FTIR evidenced the functional groups on the surfaces of the SiNPs, as described in Figure 1D. The absorption peak at 1120 cm^−1^ was attributed to the stretching vibration of the Si-O bond [50]. The strong absorption peaks of 1573 cm^−1^, 3418 cm^−1^, and 2935 cm^−1^ were assigned to the bending and the stretching vibrations of the N-H bond and the stretching vibrations of the C-H bond, respectively [51], which demonstrated the successful synthesis of SiNPs.

**Figure 2:**
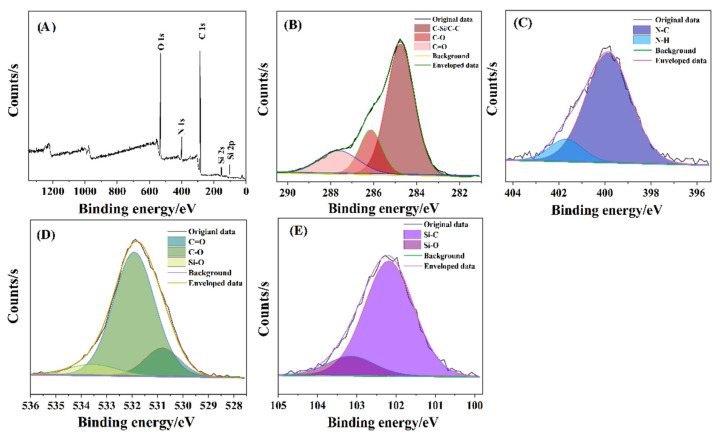
XPS profiles of SiNPs: (**A**) Survey; (**B**) C 1s; (**C**) N 1s; (**D**) O 1s; (**E**) Si 2p.

The XPS profiles further confirmed the surface composition of the SiNPs, as shown in Figure 2. The survey XPS spectrum (Figure 2A) verified that the surfaces of the SiNPs comprised C, N, O, and Si elements. In the C 1s spectrum (Figure 2B), three peaks at 285.1, 286.4, and 288.0 eV were attributed to the C-C/C-Si, C-O, and C=O bonds, respectively [52]. The peaks at 399.9 and 400.5 eV in the N1s spectrum (Figure 2C) were ascribed to the N-C and N-H bonds [53]. The three peaks in the O 1s spectrum (Figure 2D) were located at 531.0, 532.2, and 533.7 eV, which were assigned to C=O, C-O, and Si-O bonds, respectively [54]. Figure 2E shows the presence of Si-C (102.5 eV) and Si-O (103.4 eV) in the Si 2p spectrum [55]. The results of the XPS profiles are consistent with those of FTIR.

### 3.2. Optical Characterizations of SiNPs 

**Figure 3:**
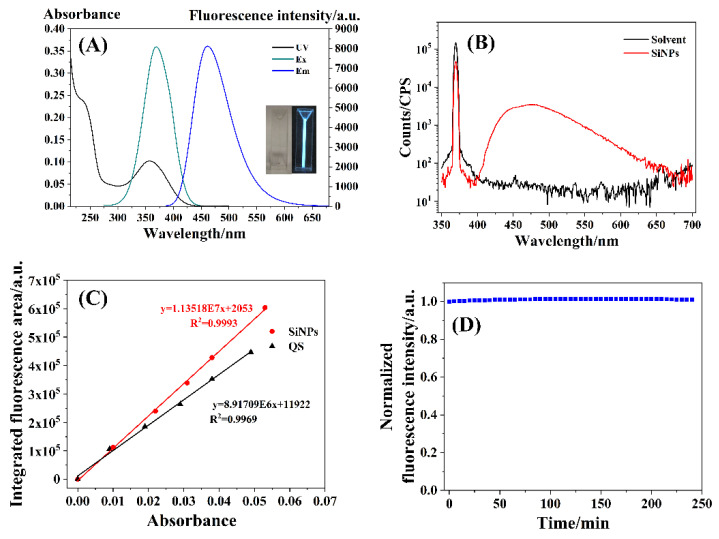
(**A**) UV–vis absorption spectrum (UV), fluorescence excitation spectrum (Ex), and fluorescence emission spectrum (Em) of the as-prepared SiNPs. Inset: the as-prepared SiNPs aqueous solution under white light (left) and 365 nm UV light (right); (**B**) the absolute fluorescence quantum yield spectra; (**C**) the relative fluorescence quantum yield spectra; (**D**) optical stability.

Figure 3 shows the optical characterizations of the resulting SiNPs. As shown in Figure 3A, the SiNPs exhibited a strong absorption peak at 360 nm, an excitation peak at 370 nm, and an emission peak at 462 nm. A possible mechanism for the fluorescence emission of SiNPs was similar to the quantum dots, which emitted the fluorescence if the size was less than its Exciton Bohr radius [56]. TEM and HRTEM images above indicated that the average size of the as-prepared SiNPs was 1.78 nm, which was less than silicon’s Exciton Bohr radius of 5 nm [57]. Hence, the synthetic SiNPs could emit fluorescence under excitation. The insets in Figure 3A show that the SiNPs solution appeared transparent under white light illumination and emitted bright blue fluorescence under UV irradiation at 365 nm. The RFQY and AFQY of the SiNPs were 73.8% and 78.9%, respectively (Figure 3B,C), proving the strong fluorescence properties of SiNPs. The results were in agreement with those reported in a previous study [58]. Figure 3D shows that the fluorescence intensity of SiNPs was unchanged after 240 min of irradiation, which demonstrated the strong fluorescence stability of the as-prepared SiNPs.

**Figure 4:**
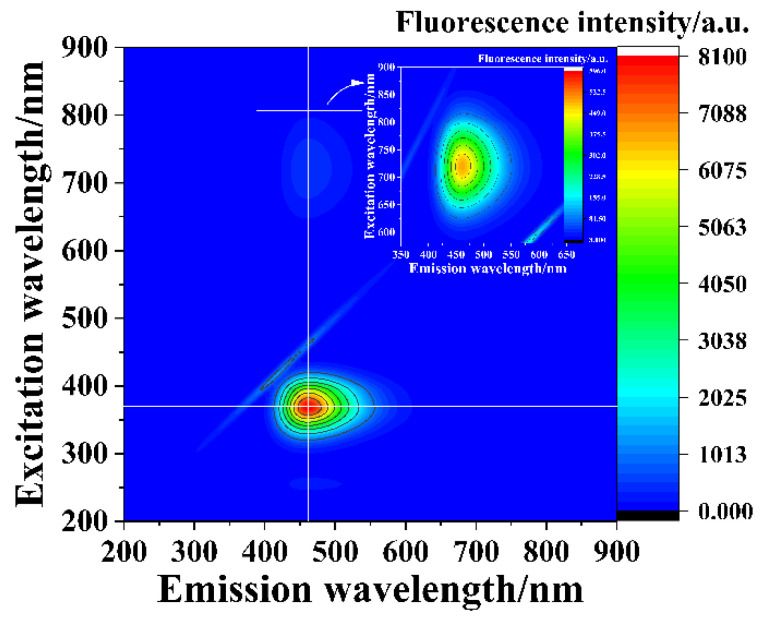
Excitation–emission map of the as-prepared SiNPs.

The emission wavelength of the as-prepared SiNPs remained almost unchanged (Figure 4) at excitation wavelengths ranging from 300 to 430 nm and 600 to 850 nm, which suggested that the as-synthesized SiNPs exhibited wavelength-independent emission behavior and excellent up-conversion fluorescence. These results were consistent with those of previous studies [59,60].

### 3.3. Detection of F^−^ Ions with the As-Prepared SiNPs

**Figure 5:**
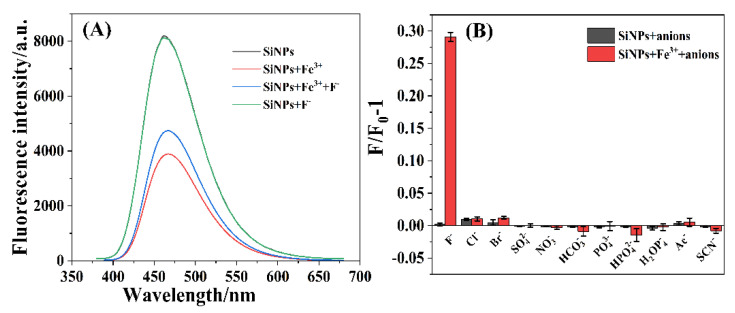
(**A**) Fluorescence spectra of SiNPs (black line), SiNPs+Fe^3+^ (red line), SiNPs + Fe^3+^ + F^−^ (blue line), and SiNPs + F^−^ (green line); (**B**) anion selectivity assays of the SiNPs and SiNPs–Fe system with anion concentration of 1 mmol·L^−1^ at 370 nm excitation.

As shown in Figure 5A, the fluorescence intensity of the SiNPs (black line in Figure 5A) firstly decreased with the addition of Fe^3+^ ions (red line in Figure 5A) and subsequently partially recovered by the further addition of F^−^ ions (blue line in Figure 5A). However, the fluorescence intensity of the aqueous solution containing SiNPs and F^−^ ions (green line in Figure 5A) was identical to that of SiNPs alone, which indicated that F^−^ ions did not influence the fluorescence of SiNPs. More than ten anions (F^−^, Cl^−^, Br^−^, SO_4_^2−^, NO_3_^−^, HCO_3_^−^, PO_4_^3−^, HPO_4_^2−^, H_2_PO_4_^−^, Ac^−^ and SCN^−^) were utilized to investigate the selectivity of the SiNPs and SiNPs–Fe system for anions. As displayed in Figure 5B, the anions scarcely influenced the fluorescence of SiNPs. Noticeably, the fluorescence intensities of the SiNPs–Fe system hardly changed in the presence of Cl^−^, Br^−^, SO_4_^2−^, NO_3_^−^, HCO_3_^−^, PO_4_^3−^, HPO_4_^2−^, H_2_PO_4_^−^, Ac^−^, and SCN^−^, except in the case of F^−^ ions (Figure 5B), which indicated the high selectivity of the SiNPs–Fe system for F^−^ ions over other anions.

**Figure 6:**
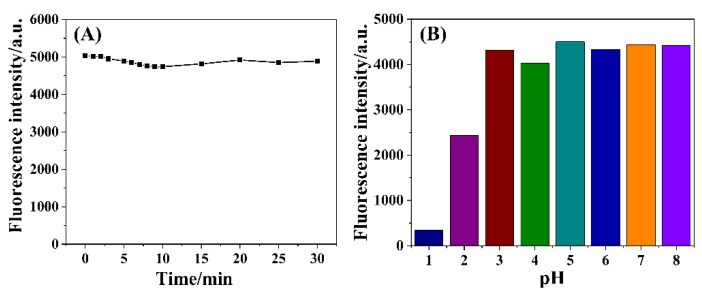
(**A**) Effect of the detection time on the fluorescence intensity; (**B**) influence of the pH on the fluorescence intensity.

Based on the fluorescence recovery after the addition of F^−^ ions into the SiNPs–Fe system, we developed a novel method for the detection of F^−^ ions. First, we investigated the effect of the detection conditions on the fluorescence intensity of the SiNPs. As shown in Figure 6A, the fluorescence intensity slightly decreased and reached a plateau approximately 10 min after adding the F^−^ ions, which indicated that a reliable detection could be achieved within 10 min. Figure 6B shows the influence of the pH on the fluorescence intensity, evidently demonstrating that the fluorescence of the SiNPs–Fe system significantly decreased at low pH values, which might have resulted from the surface structure of SiNPs–Fe system being changed in the strongly acidic environment. However, the fluorescence intensity was stable in the pH range of 5–8. Considering the aforementioned effects, Tris-HCl buffer (10 mmol·L^−1^ pH 7.2) was used to ensure a stable pH during the detection of F^−^ ions.

**Figure 7:**
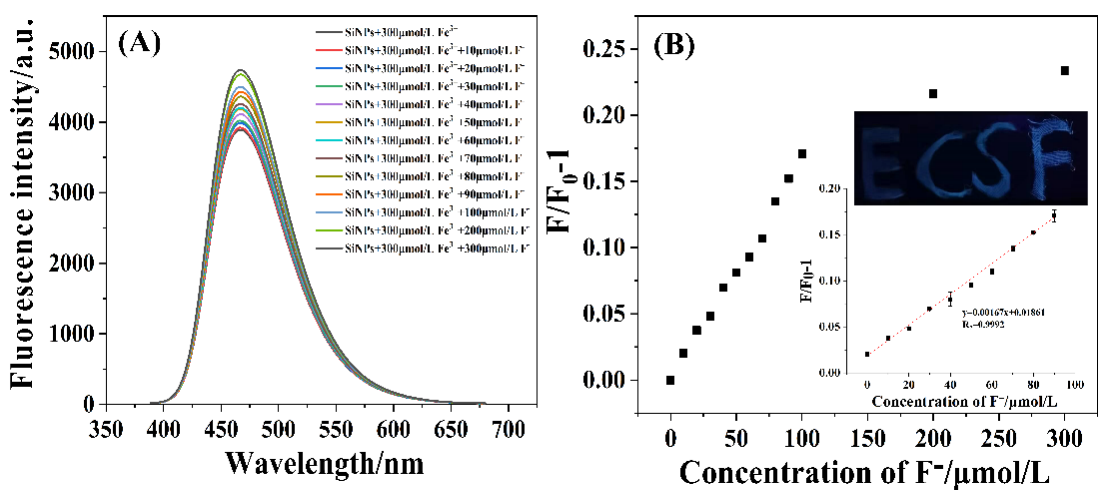
(**A**) Fluorescence spectra of as-prepared SiNPs incubated with 300 μmol·L^−1^ Fe^3+^ ions and various concentrations of F^−^ ions; (**B**) relationship between F/F_0_-1 and the concentration of F^−^ ions ranging from 0 to 300 μmol·L^−1^. Upper illustration: fluorescence images of the “E,” “C,” “S,” and “F” letters drawn using the SiNPs–Fe system in the presence of 0, 50, 100, and 200 μmol·L^−1^ F^−^ ions, respectively. Lower illustration: the linear relationship between F/F_0_-1 and F^−^ ion concentration increasing from 0 to 100 μmol·L^−1^.

Under the optimal detection conditions, the fluorescence intensities of the SiNPs–Fe system gradually increased as the concentration of F^−^ increased from 0 to 300 μmol·L^−1^ (Figure 7A). Figure 7B shows the relationship between F/F**_0_**-1 (where F_0_ and F refer to the fluorescence intensities of the SiNPs–Fe system in the absence and presence of F^−^ ions, respectively) and the concentrations of F^−^ ions in the range of 0–300 μmol·L^−1^. In the upper illustration of Figure 7B, the letters of “E,” “C,” “S,” and “F” refer to the SiNPs–Fe system incubated with 0, 50, 100, and 200 μmol·L^−1^ F^−^ ions for 10 min, respectively. The gradual changed in the brightness of the letters resulted from the fluorescence recovery of the SiNPs–Fe system depending on the F^−^ ion concentration. The lower illustration in Figure 7B shows a good linear relationship (R^2^ = 0.9992) between F/F_0_-1 and the concentrations of F^−^ ions varying from 0 to 100 μmol·L^−1^. The limit of detection (LOD) for F^−^ was found to be 0.05 μmol·L^−1^ (S/N = 3). Table 1 summarizes the previously reported detection methods using distinct fluorescence nanoparticles, which showed that the described method was comparable with those reported in other studies.

### 3.4. Fluorescence Recovery Mechanism between SiNPs–Fe System and F^−^ Ions

**Figure 8:**
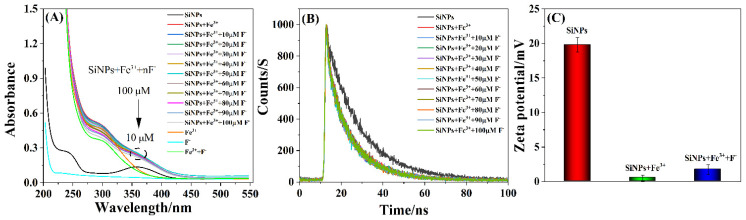
(**A**) UV–vis absorption spectra, (**B**) the fluorescence decay curves, and (**C**) the zeta potentials of the SiNPs, SiNPs+Fe^3+^, and SiNPs+Fe^3+^+F^−^ systems.

The fluorescence recovery mechanism of the SiNPs–Fe system in the presence of F^−^ ions was investigated by UV–vis absorption spectra, fluorescence decay, and zeta potential measurements. As shown in Figure 8A, there was no obvious absorption peak of F^−^ ions (blue-green line in Figure 8A), and the absorption peaks of SiNPs (black line in Figure 8A) and aqueous Fe^3+^ ions (yellow line in Figure 8A) were located at 360 and 300 nm, respectively. Figure S1 displays the UV absorption spectra of SiNPs, Fe^3+^ ion, as well as the excitation (Ex) and emission (Em) spectra of SiNPs. The UV absorption spectrum of Fe^3+^ ions (red line) slightly overlapped with the UV absorption spectrum (black line) and the excitation spectrum (green line) of SiNPs, which was likely to cause the excitation of SiNPs to be weakened. Therefore, the possible fluorescence quenching mechanisms between SiNPs and Fe^3+^ ions may originate from electron transfer [65]. Additionally, the solutions of the SiNPs–Fe system turned red with the Fe^3+^ concentration increasing from 0 to 50 μmol·L^−1^ in the presence of 1,10-Phenanthroline (Figure S2a,b), which indicated the formation of Fe^2+^ ions, further demonstrating that electron transfer was responsible for the fluorescence quenching mechanism of SiNPs and Fe^3+^ ions. Meanwhile, the SiNPs–Fe system (red line in Figure 8A) exhibited two absorption peaks at 360 and 300 nm, which were attributed to SiNPs and Fe^3+^ ions, respectively. The absorbance of the SiNPs–Fe system at 360 nm was almost invariant with an increase in the F^−^ ion concentration from 0 to 100 μmol·L^−1^, while, at the same time, the absorbance at 300 nm decreased, which indicated that the presence of F^−^ ions affected the Fe^3+^ ions rather than the SiNPs, probably due to the formation of stable colorless compounds [FeF_6_]^3−^ [66]. In addition, the absorbance of the SiNPs–Fe system at 300 nm in the presence of 100 μmol·L^−1^ of F^−^ ions (gray line in Figure 8A) was nearly equivalent to that of the solution containing Fe^3+^–F^−^ ions (green line in Figure 8A), which further indicated the presence of the colorless [FeF_6_]^3−^. Figure 8B displays the fluorescence decay curves, which further evidence the interaction between SiNPs, Fe^3+^ ions, and F^−^ ions. The fluorescence lifetime of the SiNPs decreased with the addition of Fe^3+^ ions, but did not recover with the subsequent addition of F^−^ ions, which suggested a dynamic quenching between SiNPs and Fe^3+^ ions [67]. The zeta potential of the SiNPs was 19.8 mV and drastically declined to 0.493 mV upon the addition of Fe^3+^ ions, but slightly increased to 1.72 mV with the subsequent addition of F^−^ ions, which was attributed to the interaction between F^−^ ions and Fe^3+^ ions on the surfaces of the SiNPs to restore the zeta potential of the SiNPs–Fe system (Figure 8C).

### 3.5. Detection of F^−^ Ions in Real Samples

Tap water, Antarctic krill, and Antarctic krill powder were used to evaluate the accuracy and reliability of the as-synthesized SiNPs to detect F^−^ ions in real samples. The F^−^ ion content in tap water was 21.90 μmol·L^−1^, measured using the established fluorescence (FL) method (the first column in Table 2), which was close to the value of 26.31 μmol·L^−1^ measured using a fluoride ion selective electrode (FISE, the last column in Table 2) method, thus verifying the accuracy of the proposed method. The recoveries of the standard addition assays were 87.8%, 110.2%, and 102.2% for 20, 50, and 70 μmol·L^−1^ spiked concentrations of F^−^ ions in tap water, respectively, with the relative standard deviations (RSDs) of 9.65%, 6.08%, and 2.68%, respectively, which further demonstrated the accuracy and feasibility of the developed method. The levels of water-soluble F^−^ ions were measured to be 196 and 23.0 mg·kg^−1^ in the Antarctic krill and Antarctic krill powder by the proposed method, respectively, which were almost consistent with those of 199 and 22.4 mg·kg^−1^ detected by the FISE method, respectively. The results confirmed the accuracy and reliability of the as-synthesized SiNPs for the detection of F^−^ in real samples. According to the average percentages of 15% (the water-soluble fluorine vs. the total fluorine [68]), the fluorine content was high, at 1300 mg·kg^−1^ in the Antarctic krill. Nevertheless, the fluorine content of human intake would not exceed the standard of 100 mg·kg^−1^ based on the regulations of the USA Food and Drug Administration (FDA). Hence, Antarctic krill cannot be eaten directly as food, and needs to be pre-treated (e.g., defluorination) before eating.

## 4. Conclusions

We developed a facile and feasible method for the detection of F^−^ ions based on a novel fluorescence probe with excellent optical silicon nanoparticles (SiNPs). The fluorescence of SiNPs was quenched in the presence of Fe^3+^ ions and restored upon the addition of F^−^ ions. The recovery of fluorescence intensity linearly depended on the F^−^ concentration varying from 0 to 100 μmol·L^−1^ (R^2^ = 0.9992) with a detection limit of 0.05 μmol·L^−1^. UV–vis absorption spectra, fluorescence decay curves, and zeta potential measurements verified the fluorescence recovery mechanism via the formation of a stable and colorless ferric fluoride compound. Moreover, the SiNPs were successfully used to detect F^−^ ions in tap water and food samples. The results demonstrated the accuracy and reliability of the established method based on SiNPs.

## Figures and Tables

**Table 1:** Comparison employing fluorescent probes to detect F^−^ ions.

Probe	Linear Range/μmol·L^−1^	LOD/μmol·L^−1^	Ref.
Organic fluorescent substances	0–20	-	[61]
Zr(H_2_O)_2_EDTA@CDs	0.1–10	-	[62]
Fe_3_O_4_@SiO_2_@CDs	1–20	0.06	[63]
N-CDs	0–50	0.059	[64]
SiNPs	0–100	0.05	This work

**Table 2:** Standard addition assays based on fluorescent SiNPs in tap water.

Real Samples	Initial Amount/μmol·L^−1^	Spiked/μmol·L^−1^	Found/μmol·L^−1 a^	RSDs/%	Recovery/%	Initial Amount/μmol·L^−1^
Tap water	21.90 (FL)	20	24.86 ± 2.40	9.65	87.8	26.31 (FISE)
		50	62.41 ± 3.79	6.08	110.2	
		70	78.84 ± 2.11	2.68	102.2	

^a^ The average standard deviation was calculated from three parallel measurements.

## Data Availability

Data sharing is not applicable.

## Supplementary Materials

The following are available online at https://www.mdpi.com/article/10.3390/nano12020213/s1, Figure S1: The UV absorption spectra of SiNPs, Fe^3+^ ion, as well as the excitation (Ex) and the emission (Em) spectra of SiNPs, Figure S2: The images of SiNPs and Fe^3+^ with the concentration increasing from 0 to 50 μmol·L^−1^ in the absence (**a**) and the presence (**b**) of 1,10-Phenanthroline.
