# Supplementary material for: “Switch-Off-On” Detection of Fe3+ and F− Ions Based on Fluorescence Silicon Nanoparticles and Their Application to Food Samples
Source: Nanomaterials (Basel). 2022 Jan 10;12(2):213. doi: 10.3390/nano12020213 (PMC8779261; doi:10.3390/nano12020213)
Supplement: Supplementary file 1 [file nanomaterials-12-00213-s001.zip › nanomaterials-1487382-supplementary.pdf]

nanomaterials

Supporting Information

"Switch-Off-On" Detection of Fe^{3+} and F^{-} Ions Based on Fluorescence Silicon Nanoparticles and Their Application to Food Samples

Hongli Ye [1,2,†], Lukai Zhao [3,†], Xinghui Ren [4], Youqiong Cai [1,2] and Hai Chi [1,3,*]

[1] Laboratory of Aquatic Product Quality, Safety and Processing, East China Sea Fisheries Research Institute, Chinese Academy of Fishery Sciences, Shanghai 200090, China; yehongli12@163.com (H.Y.); caiyouqiong@163.com (Y.C.); andychihai@126.com
[2] Key Laboratory of Control of Safety and Quality for Aquatic Product, Ministry of Agriculture and Rural Affairs, Beijing 100141, China
[3] School of Medical Instrument and Food Engineering, University of Shanghai for Science and Technology, Shanghai 200093, China; 13962892027@163.com
[4] State Key Laboratory of Medicinal Chemical Biology, Tianjin Key Laboratory of Biosensing and Molecular Recognition, Research Center for Analytical Sciences, College of Chemistry, Nankai University, Tianjin 300071, China; 1120200313@mail.nankai.edu.cn
* Correspondence: andychihai@126.com
† These authors contributed equally to this work.

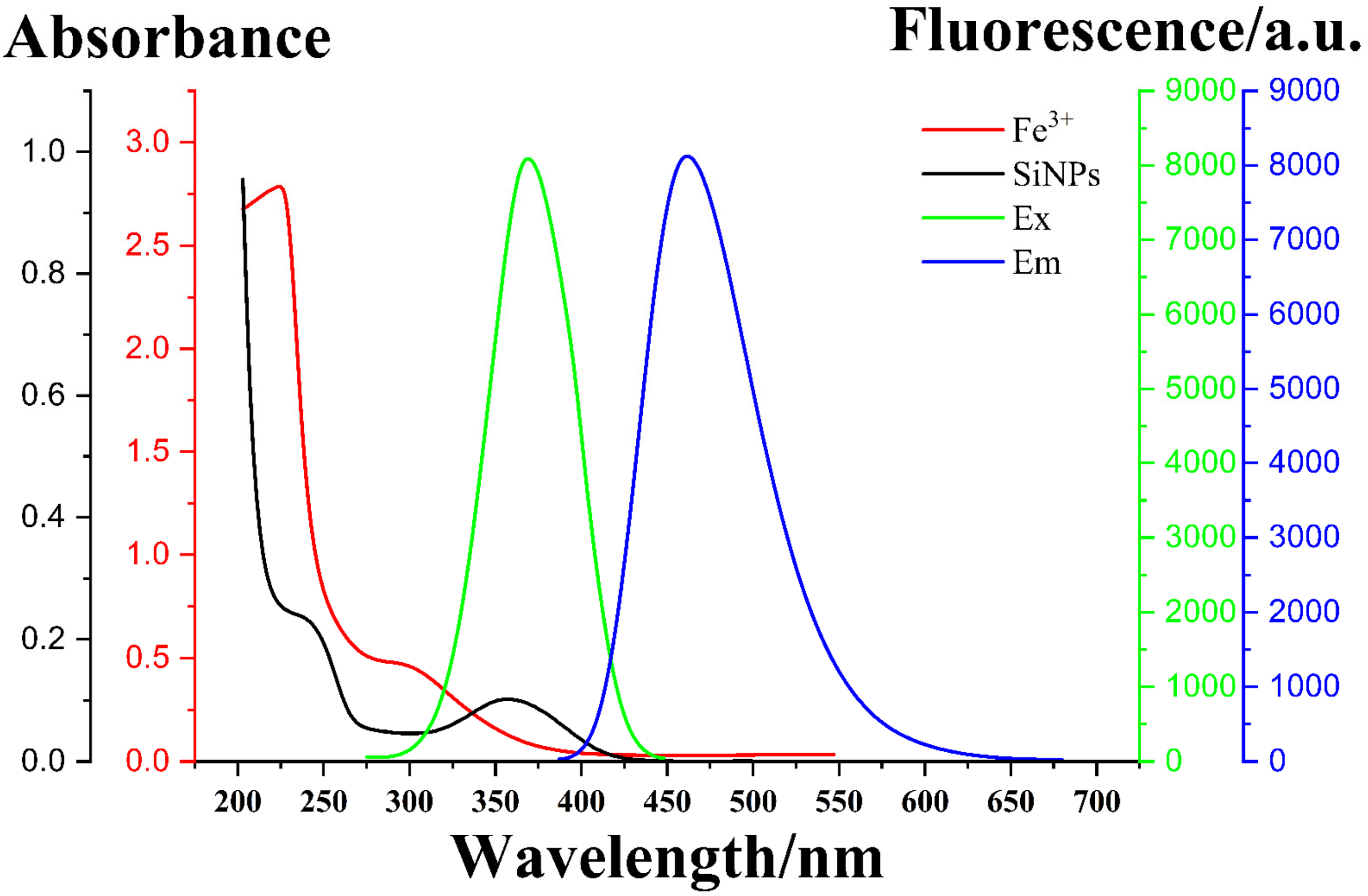

Figure S1. The UV absorption spectra of SiNPs, Fe^{3+} ion, as well as the excitation (Ex) and the emission (Em) spectra of SiNPs.

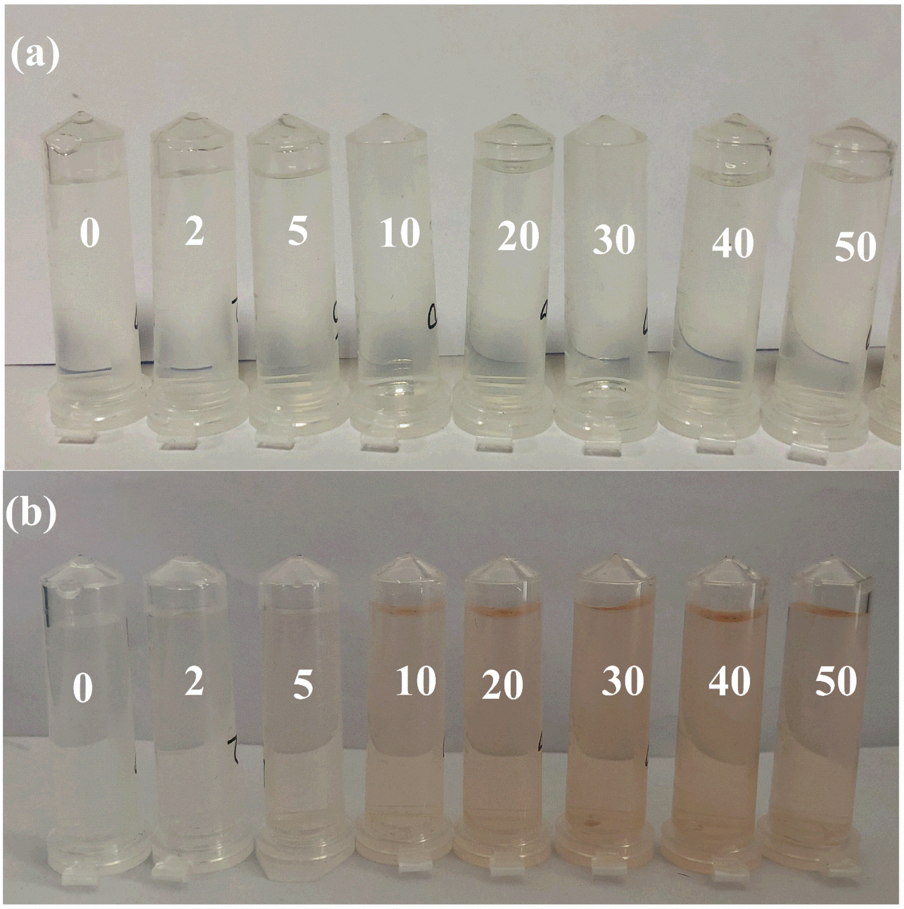

Figure S2. The images of SiNPs and Fe^{3+} with the concentration increasing from 0 to 50 μmol·L^{-1} in the absence (**a**) and the presence (**b**) of 1,10-Phenanthroline.
